# Critical role of histone demethylase RBP2 in human gastric cancer angiogenesis

**DOI:** 10.1186/1476-4598-13-81

**Published:** 2014-04-09

**Authors:** Lupeng Li, Lixiang Wang, Ping Song, Xue Geng, Xiuming Liang, Minran Zhou, Yangyang Wang, Chunyan Chen, Jihui Jia, Jiping Zeng

**Affiliations:** 1 Department of Biochemistry and Molecular Biology, Shandong University School of Medicine, Jinan 250012, P. R. China; 2 Department of Pharmacology, Shandong University School of Medicine, Jinan 250012, P. R. China; 3 Department of Microbiology/Key Laboratory for Experimental Teratology of Chinese Ministry of Education, Shandong University School of Medicine, Jinan 250010, P. R. China; 4 Department of Hematology, Qilu Hospital, Shandong University School of Medicine, Jinan 250012, P.R. China

**Keywords:** RBP2, VEGF, Gastric cancer, Angiogenesis

## Abstract

**Background:**

The molecular mechanisms responsible for angiogenesis and abnormal expression of angiogenic factors in gastric cancer, including vascular endothelial growth factor (VEGF), remain unclear. The histone demethylase retinoblastoma binding protein 2 (RBP2) is involved in gastric tumorgenesis by inhibiting the expression of cyclin-dependent kinase inhibitors (CDKIs).

**Methods:**

The expression of RBP2, VEGF, CD31, CD34 and Ki67 was assessed in 30 human gastric cancer samples and normal control samples. We used quantitative RT-PCR, western blot analysis, ELISA, tube-formation assay and colony-formation assay to characterize the change in VEGF expression and associated biological activities induced by RBP2 silencing or overexpression. Luciferase assay and ChIP were used to explore the direct regulation of RBP2 on the promoter activity of VEGF. Nude mice and RBP2-targeted mutant mice were used to detect the role of RBP2 in VEGF expression and angiogenesis *in vivo*.

**Results:**

RBP2 and VEGF were both overexpressed in human gastric cancer tissue, with greater microvessel density (MVD) and cell proliferation as compared with normal tissue. In gastric epithelial cell lines, RBP2 overexpression significantly promoted the expression of VEGF and the growth and angiogenesis of the cells, while RBP2 knockdown had the reverse effect. RBP2 directly bound to the promoter of VEGF to regulate its expression by histone H3K4 demethylation. The subcutis of nude mice transfected with BGC-823 cells with RBP2 knockdown showed reduced VEGF expression and MVD, with reduced carcinogenesis and cell proliferation. In addition, the gastric epithelia of RBP2 mutant mice with increased H3K4 trimethylation showed reduced VEGF expression and MVD.

**Conclusions:**

The promotion of gastric tumorigenesis by RBP2 was significantly associated with transactivation of VEGF expression and elevated angiogenesis. Overexpression of RBP2 and activation of VEGF might play important roles in human gastric cancer development and progression.

## Background

Gastric cancer is an important health problem around the world and the cause of 12% of all cancer-related deaths each year [1,2], especially in China, which accounts for 42% of the global total cases [3]. Carcinogenesis is a multistep process involving the transformation, survival, proliferation, invasion, angiogenesis, and metastasis of tumor. During this process, the accumulation of genetic and epigenetic alterations leads to progressive transformation [4]. Determining the molecular mechanism can benefit diagnosis and treatment.

Angiogenesis is a process of neovascular formation from pre-existing blood vessels, which consists of sequential steps for vascular destabilization, angiogenic sprouting, lumen formation and vascular stabilization. Induction of angiogenesis represents one of the major hallmarks of cancer [5]. Vascular endothelial growth factor (VEGF) is a key mediator in the neovascularization of cancers [6]. Through a VEGF-induced signaling pathway, such as PI3K-AKT, JAK2-STAT5 and ERK1/2, VEGF is involved in angiogenesis and proliferation [7]. VEGF overexpression involves abnormal expression of growth factors and their receptors [8]. Epigenetic regulation is important for VEGF expression. VEGF expression is suppressed by promoter hypermethylation [9]. MiR-125a and miR-126 can inhibit its expression [10,11]. To better understand the mechanism of angiogenesis, the regulation of VEGF expression is a key point.

Histone modification plays an important role in carcinogenesis, including angiogenesis [5,12]. For example, histone methylase MLL1 has critical roles in tumor growth and angiogenesis [13]. HDAC3 acts as a negative regulator of angiogenesis [14]. Retinoblastoma binding protein 2 (RBP2), a newly found histone demethylase for H3 lysine 4 (H3K4) trimethylation and dimethylation, is involved in gastric tumorgenesis with its epigenetic inhibition of cyclin-dependent kinase inhibitors (CDKIs) [15]. We also found a high expression of RBP2 in hepatocellular carcinoma and its inhibition triggered cell senescence [16]. Others also suggested that loss of RBP2 suppresses tumorigenesis in mice lacking Rb1 or Men1 [17].

In the present study, we sought to determine the potential role of RBP2 in human gastric cancer angiogenesis via the regulation of VEGF and the underlying mechanisms *in vivo* and *in vitro*.

## Results

### Association of RBP2 overexpression with increased VEGF expression and MVD in human gastric cancer

**Figure 1:**
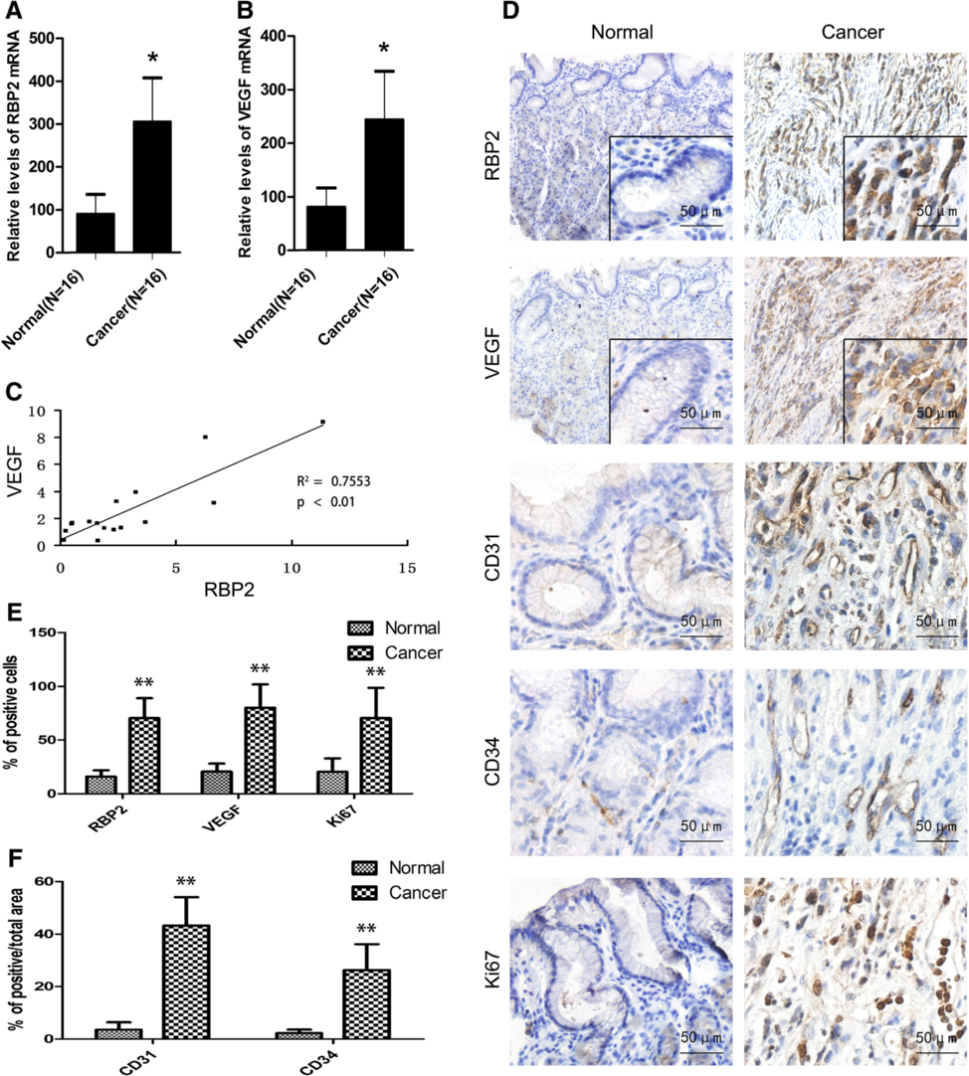
**Association of RBP2 overexpression and increased VEGF expression in human gastric cancer.** QRT-PCR analyses of **(A)** RBP2 mRNA and **(B)** VEGF mRNA in normal and cancerous human gastric tissues. *P < 0.05. **(C)** Correlation of RBP2 and VEGF levels in human gastric cancer tissues after standardization with matched normal tissues. **P < 0.01. **(D)** Immunohistochemical staining of expression of RBP2, VEGF, CD31, CD34 and Ki67 in human normal (left panel) and cancerous (right panel) gastric tissues. Representative images are shown. **(E)** Percentage positive cells by immunohistochemistry for RBP2, VEGF and Ki67 in human normal and cancerous gastric tissues. **P < 0.01. **(F)** Percentage positive area by immunohistochemistry for CD31 and CD34 in human normal and cancerous gastric tissues. **P < 0.01. Data are mean ± SEM of 3 independent experiments.

As compared with normal human tissues, gastric cancer specimens showed overexpression of RBP2 and VEGF mRNA and the correlation of mRNA expression, which supports the role of RBP2 and VEGF in tumorgenesis and their co-expression *in vivo* (Figure 1A, B and C). By Immunohistochemistry (IHC) we observed high RBP2 expression in 14 cases (70%) and low expression in 6 cases (30%). At the same sites, we observed strong VEGF expression in 15 cases (75%) and weak expression in 5 cases (25%). In addition, we observed high MVD in 15 cases (75%) and low MVD in 5 cases (25%) as seen by positive CD31 and CD34 staining (Figure 1F). The expression pattern of RBP2 was consistent with that of VEGF and MVD status. The marker of cell proliferation, Ki67, was also overexpressed in human gastric cancer specimens (Figure 1D, E). This clinical evidence supports the association of RBP2 and VEGF expression and increased angiogenesis in gastric cancer. RBP2 or VEGF expression was associated with tumor size but not age, gender, specimen histology or differentiation (Additional file 1: Table S1).

### Regulation of VEGF expression by RBP2 in human gastric cancer cells and RBP2-targeted mutant mice

**Figure 2:**
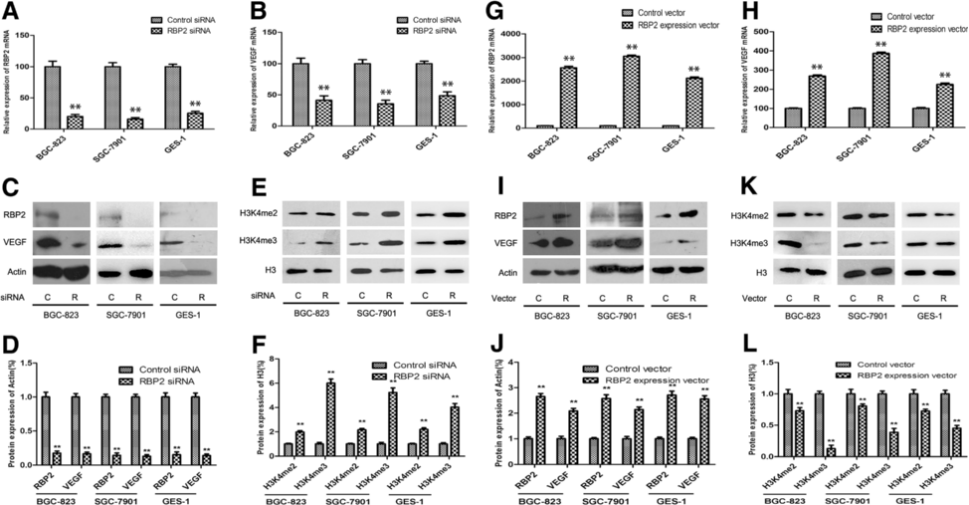
**The direct regulation of VEGF by RBP2 in human gastric cancer cells.** QRT-PCR analyses of **(A)** RBP2 and **(B)** VEGF mRNA level in control and RBP2 siRNA (10 nM)-transfected BGC-823, SGC-7901 and GES-1 cell lines after 72 h. **P < 0.01. Western blot analyses of **(C, D)** RBP2 and VEGF protein levels and **(E, F)** tri- and di- methylation of H3K4 in cancer cells treated with control and RBP2 siRNAs. C and R: Control and RBP2 siRNA, respectively. **P < 0.01. QRT-PCR analyses of **(G)** RBP2 and **(H)** VEGF mRNA levels in cancer cells transfected with control and RBP2 expression vector (4 μg) after 48 h. **P < 0.01. **(I, J)** Western blot analyses of RBP2 and VEGF protein levels and **(K, L)** tri- and di- methylation of H3K4 in cancer cells treated with control and RBP2 expression vectors. Vector C and R: Control pcDNA3.1 and pcDNA3.1-RBP2 expression plasmids, respectively. **P < 0.01. Data are mean ± SEM of 3 independent experiments.

To obtain direct evidence of whether RBP2 regulated the expression of VEGF and was involved in the angiogenesis of gastric cancer, we transfected RBP2 expression vector and siRNA into BGC-823, SGC-7901 and GES-1 cells. Cells transfected with RBP2 siRNA showed decreased mRNA and protein levels of VEGF as compared with control siRNA transfection (Figure 2A-D). As well, histone H3K4 tri- and dimethylation was increased with RBP2 silencing (Figure 2E and F). In contrast, cells with RBP2 overexpression showed increased mRNA and protein expression of VEGF as compared with control transfection (Figure 2G-J). Histone H3K4 tri- and dimethylation was decreased with overexpression of RBP2 (Figure 2K and L).

**Figure 3:**
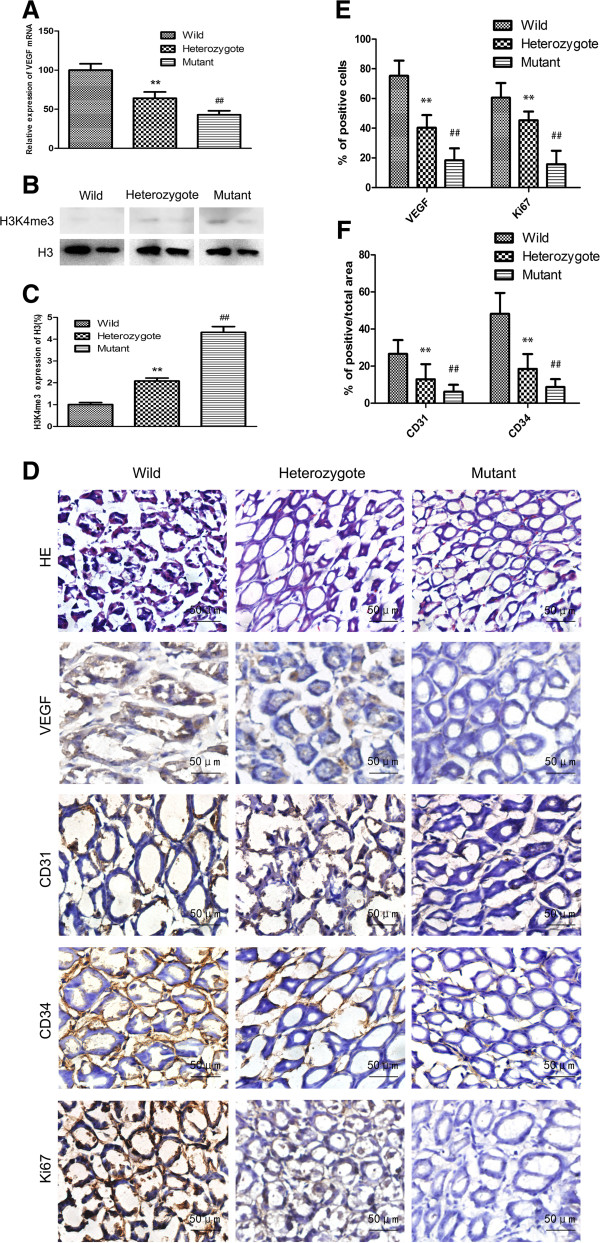
**VEGF expression and angiogenesis in RBP2-targeted mutant mice. (A)** QRT-PCR analysis of VEGF mRNA in the wild type (the number is 6), heterozygote (4) and mutant mice (3). **P < 0.01 *vs* wild type group, ##*P* < 0.01 *vs* wild type group. **(B, C)** Trimethylation of H3K4 in wild-type, heterozygote and mutant mice. Two representatives of each group are shown. **P < 0.01 *vs* wild type group, ##*P* < 0.01 *vs* wild type group. **(D)** Hematoxylin and eosin (HE) staining and immunohistochemical staining of VEGF, CD31, CD34 and Ki67 expression in wild-type (left panel), heterozygote (middle panel) and mutant mice (right panel). Representative images are shown. Percentage positive cells for **(E)** VEGF and Ki67 and **(F)** percentage positive staining area for CD31 and CD34 in wild-type, heterozygote and mutant mice determined immunohistochemically. **P < 0.01 *vs* wild type group, ##*P* < 0.01 *vs* wild type group. Data are mean ± SEM of 3 independent experiments.

Furthermore, to determine whether the regulation of VEGF expression and angiogenesis coexisted with H3K4 demethylation of RBP2, we divided RBP2-targeted mutant mice into mutant controls, heterozygotes and wild types (Additional file 2: Figure S1) and examined VEGF expression and MVD in gastric epithelia from the 3 groups. As compared with the wild type, mutant and heterozygote groups showed inhibited VEGF expression and enhanced H3K4 trimethylation with RBP2 mutation (Figure 3A-E) as well as inhibited MVD as revealed by CD31 and CD34 staining (Figure 3D and F). The expression pattern for Ki67 was similar (Figure 3D and E). Blockade of RBP2 demethylase activity may have suppressed VEGF expression and impaired gastric angiogenesis.

### Transcriptional activation of VEGF expression in gastric cancer cells by RBP2

**Figure 4:**
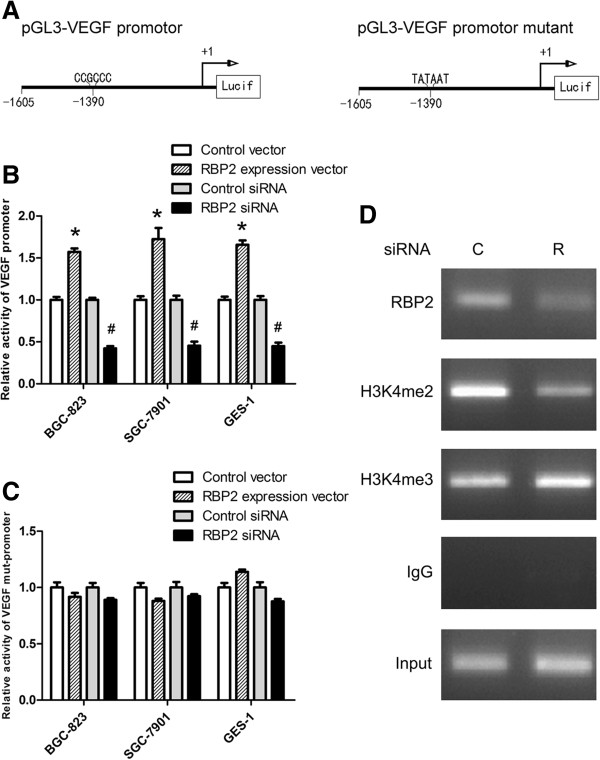
**Transactivation of VEGF promoter induced by RBP2. (A)** Schematic structure of pGL3-VEGF promoter and pGL3-VEGF promoter mutant. **(B)** After transfection of RBP2 expression vector for 48 h, luciferase activity of VEGF promoter reporters as compared with the control vector and RBP2 siRNA cotransfection for 72 h as compared with control siRNA. *P < 0.05 *vs* control vector. #*P* < 0.05 *vs* control siRNA. **(C)** The luciferase activity of VEGF promoter mutant reporters with RBP2 expression vector and RBP2 siRNA cotransfection. **(D)** RBP2 occupancy and trimethylation status of H3K4 at the promoter of the VEGF gene in BGC-823 cells. C and R: Control siRNA and RBP2 siRNA, respectively. Data are mean ± SEM of 3 independent experiments.

Because VEGF expression was regulated by RBP2 at both mRNA and protein levels, RBP2 might bind to the promoter of VEGF directly. To determine whether RBP2 regulated VEGF promoter activity, we cotransfected pGL3-VEGF or pGL3-VEGF-mutant (Figure 4A) into BGC-823, SGC-7901 and GES-1 cells with RBP2 siRNA or control siRNA and RBP2 expression vector or control vector. Cotransfection with RBP2 expression vector activated luciferase activity driven by the VEGF promoter (Figure 4B). Conversely, inhibition of RBP2 expression decreased the luciferase activity driven by the VEGF promoter. Mutations of the putative RBP2 binding site attenuated the change in luciferase activity (Figure 4B and C). To determine whether VEGF was the direct target of RBP2, we performed ChIP assay to determine an association of RBP2 with the VEGF promoter. In cells treated with control siRNA, RBP2 occupancy on the promoter region of VEGF gene was readily detectable. In contrast, knocking down RBP2 abolished the association with this promoter sequence (Figure 4D). Consistent with its H3K4 demethylase activity, RBP2 depletion significantly enhanced H3-K4 trimethylation at the proximal promoter region of VEGF, as documented in Figure 4D.

### Direct effect of RBP2 on VEGF expression in the tumorigenicity of human gastric cancer cell lines

**Figure 5:**
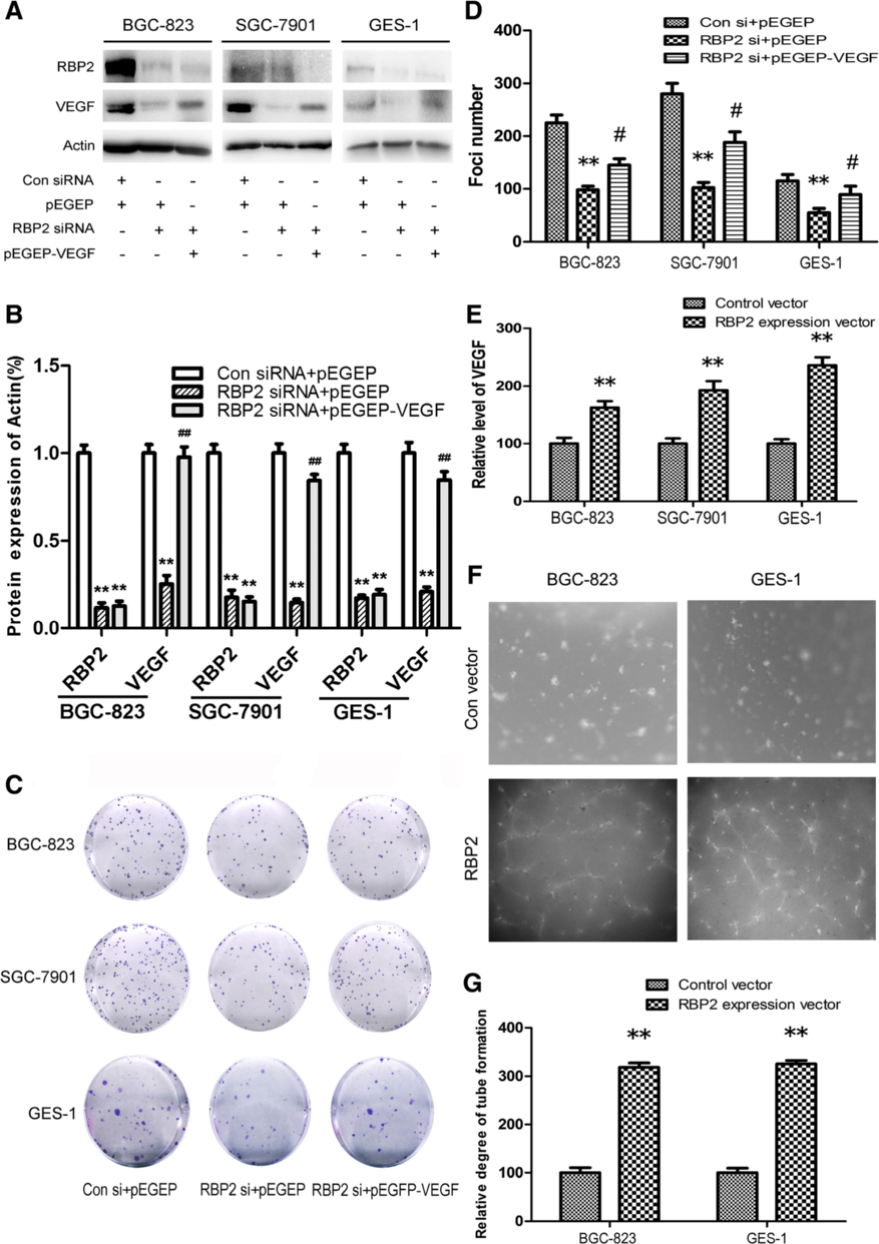
**Promotion of tumorgenesis and angiogenesis by RBP2-induced VEGF expression *in vitro*. (A, B)** Western blot analysis of RBP2 and VEGF protein levels in cancer cells treated with control and RBP2 siRNA or cotransfection of RBP2 siRNA and VEGF expression vector. **P < 0.01 *vs* control, ##*P* < 0.01 *vs* RBP2 siRNA + pEGEP. **(C)** Foci formation of human gastric cancer cells after treatment with control and RBP2 siRNA or cotransfection of RBP2 siRNA and VEGF expression vector. **(D)** The foci numbers of clones. **P < 0.01 *vs* control. #*P* < 0.05 *vs* RBP2 siRNA + pEGEP. **(E)** ELISA results for VEGF concentration in cell cultures treated with control vector and RBP2 expression vector. **P < 0.01. **(F)** Tube formation in HUVEC cells treated with the supernatants from the cells transfected with control vector and RBP2 expression vector. **(G)** Tube formation calculated as the percentage of cell surface area to the total surface area. The group incubated with the control cell cultures were set to 100%. **P < 0.01. Data are mean ± SEM of 3 independent experiments.

We used western blot analysis to determine RBP2 and VEGF expression in gastric cancer cells with RBP2 silencing or VEGF overexpression (Figure 5A and B). Clone formation assay was used to check the effect of RBP2 on VEGF expression in tumorgenesis. RBP2 siRNA in BGC-823, SGC-7901 and GES-1 significantly inhibited clone formation. Cotransfection of RBP2 siRNA and VEGF expression vector reversed this inhibition in part (Figure 5C and D).

The transfection of RBP2 expression vector markedly increased the secretion of VEGF in BGC-823, SGC-7901 and GES-1 cell supernatant (Figure 5E) and RBP2 silencing with RBP2 siRNA suppressed the VEGF secretion (Additional file 3: Figure S2). Consistent with increased expression of VEGF, the supernatants of BGC-823 and GES-1 cells with RBP2 overexpression were more angiogenic than those of control vector–transfected BGC-823 and GES-1 cells as determined by endothelial-cell tube-formation assay (Figure 5F and G). These results suggest the direct effect of RBP2 on VEGF expression in tumorgenesis and angiogenesis.

### Regulation of VEGF expression, tumorgenesis and angiogenesis by RBP2 in gastric tumors in nude mice

**Figure 6:**
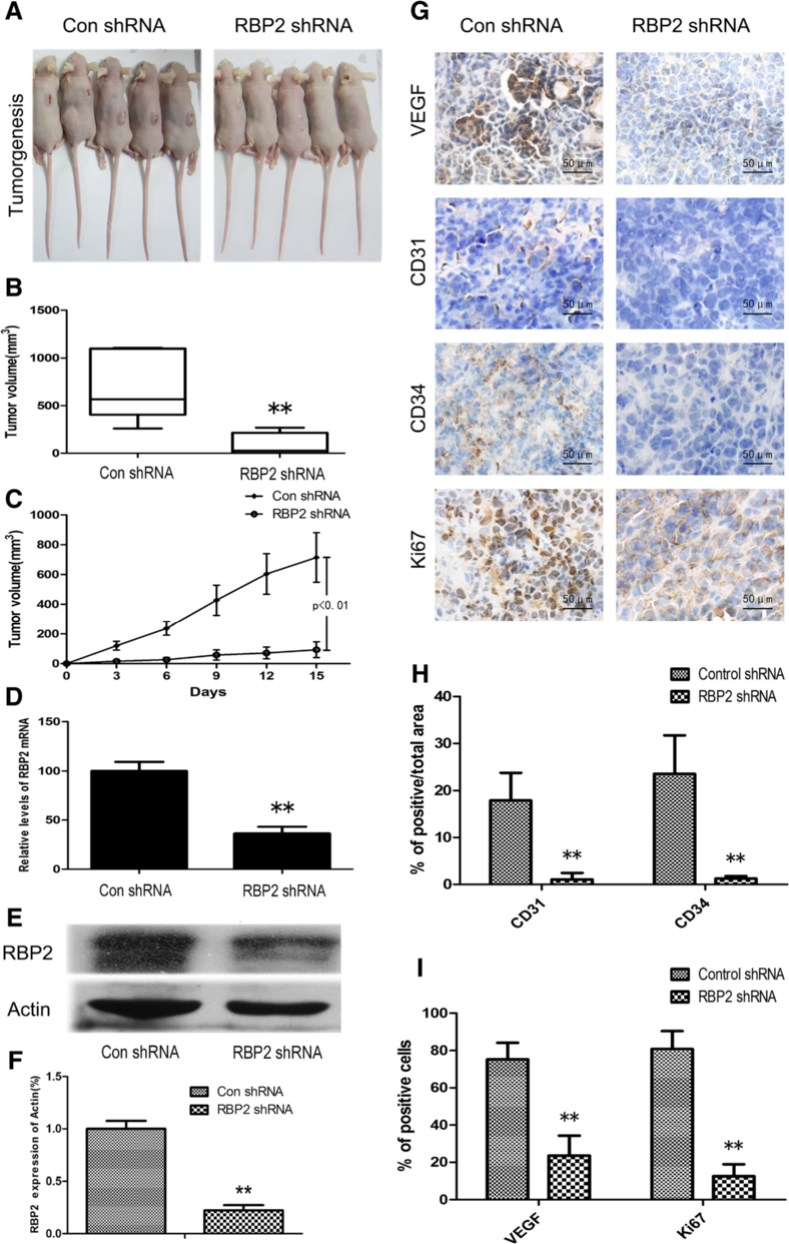
**Inhibition of VEGF expression and angiogenesis by RBP2 knockdown in nude mice. (A)** Tumorgenesis after injection of BGC-823 cells stably expressing RBP2 shRNA and control shRNA. **(B)** Tumor volume with RBP2 shRNA stable expression and controls. Data are median, with box edges as interquartile range and whiskers as the minimum and maximum values. **P < 0.01. **(C)** Growth curve with RBP2 shRNA stable expression and controls. **P < 0.01. **(D)** QRT-PCR analysis of RBP2 mRNA expression with RBP2 shRNA stable expression and controls. **P < 0.01 **(E, F)** Western blot analysis of RBP2 protein expression with RBP2 shRNA stable expression and the control. **P < 0.01. **(G)** Immunohistochemical staining for VEGF, CD31, CD34 and Ki67 in the control group (left panel) and RBP2 shRNA stable expression group (right panel). Representative images are shown. **(H)** Percentage positive area for CD31 and CD34 expression with the control and RBP2 shRNA stable expression determined immunohistochemically. **P < 0.01. **(I)** Percentage positive cells for VEGF and Ki67 with the control and RBP2 shRNA stable expression determined immunohistochemically. **P < 0.01.

We established stable lentiviral-RBP2-shRNA–transfected BGC-823 cells and injected them into the subcutis of nude mice to evaluate the effect of knockdown of RBP2 expression on gastric tumor growth and angiogenesis by regulating VEGF expression. In contrast with the large tumors produced by control cells, RBP2-shRNA–transfected BGC-823 cells produced much smaller gastric tumors with slower growth (Figure 6A, B and C). To further identify the mechanisms involved in RBP2-associated gastric tumor growth, we examined the effect of RBP2 expression on VEGF expression and tumor angiogenesis in nude mice. QRT-PCR and western blot analysis confirmed the silencing of RBP2 (Figure 6D, E and F). We identified microvessel formation by immunostaining with anti-CD31 and CD34 antibodies. Representative VEGF expression and MVD in tumors with control and RBP2-shRNA–transfected BGC-823 cells and quantification are shown in Figure 6 G, H and I. In control mice, VEGF expression and microvessel formation was greater with RBP2 overexpression than inhibition. These results suggested that alteration of tumor growth and angiogenesis with increased RBP2 expression was directly associated altered VEGF expression.

## Discussion

In this study, we focused on the critical role of the histone H3K4 demethylase RBP2 in angiogenesis of gastric cancer, especially the direct regulation of VEGF by RBP2. First, we determined the association of RBP2 expression, VEGF expression and MVD status in human gastric cancer tissue species. Second, we detected the direct regulation of VEGF by RBP2 both in human gastric cancer cell lines and RBP2-targeted mutant mice, which showed that H3K4 demethylation by RBP2 expression was important for VEGF expression and MVD status. RBP2 bound to the promoter of VEGF directly and activated the promoter of VEGF for regulation at the transcriptional level. Third, VEGF expression induced by RBP2 overexpression activated tumorigenesis and angiogenic potential in human gastric cancer cells and animal models, whereas silencing of RBP2 expression had the reverse effect. Therefore, abnormal RBP2 expression and VEGF activation might explain the poor prognosis with gastric cancer and contribute directly to gastric tumor angiogenesis and aggressive gastric cancer biology.

Angiogenesis is a critical process in the invasion, growth and metastasis of most solid tumors [18]. Angiogenesis is complex and involves a large number of molecules. VEGF, fibroblast growth factor, angiopoietin, Notch, transforming growth factor β, Hedgehog and WNT signaling cascades orchestrate angiogenesis through the direct or indirect regulation of quiescence, migration and the proliferation of endothelial cells [19-23]. VEGF has critical roles in tumor angiogenesis [24]. Small-molecule compounds and human/humanized monoclonal antibodies interrupting VEGF signaling have been developed as anti-angiogenic therapeutics for cancer [25,26]. Epigenetic regulation in angiogenesis is a new pathway in carcinogenesis and metastasis. Lysine acetylation and cytosine methylation are important transcriptional regulators of angiogenic genes in endothelial cells. Lysine acetylation and cytosine methylation inhibitors idiosyncratically tune the transcriptome and affect expression of key modulators of angiogenesis such as VEGF and endothelial nitric oxide synthase [27].

We have found that RBP2, a new histone H3K4 demethylase, is involved in carcinogenesis by escaping cell senescene and inhibiting CDKIs [15,16]. In this research, we first found RBP2 activation associated with VEGF expression and tumorgenesis and angiogenesis in human gastric cancer: RBP2 expression was positively associated with VEGF mRNA and protein expression and with MVD as identified by CD31 and CD34 antibody staining and cell proliferation by Ki67 overexpression, which suggests a link between RBP2 activation and VEGF overexpression. RBP2 overexpression significantly affected VEGF expression in gastric cancer cells via demethylation of H3K4. In RBP2-targeted mutant mice with H3K4 demethylation of RBP2 silenced and H3K4 trimethylation enhanced, the expression of VEGF and the aniogenic phenotype were also inhibited. As well, RBP2 expression was involved in VEGF promoter activity in gastric cancer cells. Specifically, we identified one potential RBP2 binding site on the VEGF promoter. Mutation of the site profoundly attenuated but did not completely eliminate RBP2-mediated transactivation of the VEGF promoter. Therefore, we confirmed by ChIP assay that this binding site was functional, which showed active recruitment of RBP2 to the binding site.

H3-K4 methylation is associated with transcription activation [28-30]. As a histone demethylase specific for di- and tri-methylated H3-K4, RBP2 acts as a transcriptional repressor by inhibiting H3-K4 methylation at its target promoters [31,32]. However, we found that VEGF was a direct target of RBP2. Not as the suppression of CDKIs promoters by RBP2 in gastric cancer, RBP2 induced the activation of VEGF promoter by directly binding to the CCGCCC DNA motif [33]. Another important H3K4 demethylase, LSD1, which is overexpressed in numerous cancers, was found involved in inducing VEGF expression in prostate cancer [34]. LSD1 was also identified to act through its demethylase activity to promote epigenetic modifications at Notch-target genes. Remarkably, LSD1 functions as a corepressor when associated with the CSL-repressor complex and as a NOTCH1 coactivator upon Notch activation [35]. In addition, RBP2 is present in a number of chromatin-remodeling complexes [36,37]. Polycomb-repressive complex 2 recruits RBP2 to its target genes via physical interaction [32]. This situation may occur with the promoter of the VEGF gene. However, we need more evidence to determine whether RBP2 affects the VEGF promoter methylation status, which is involved in the regulation of VEGF expression [9] in human gastric cancer cells.

We detected the critical role of RBP2 in angiogenesis by the biological effect in human cancer cells and animal models. With RBP2 siRNA, RBP2 expression was silenced and VEGF expression inhibited. The clone formation of human gastric cancer cells was also suppressed in this process, which was partially reversed by the overexpression of VEGF in RBP2 siRNA-transfected cells. We found RBP2 involved in gastric carcinogenesis by the regulation of VEGF. In nude mice, tumors from transfected gastric cancer cells stably expressing RBP2 shRNA were smaller, with lower VEGF expression, and less MVD and cell proliferation than control cells. Recent genome-wide analyses of mouse embryonic stem cells and human leukemic cell lines revealed hundreds of RBP2 target genes and many of them implicated in development, proliferation and differentiation controls [32,38,39]. RBP2 was identified as a key molecule in drug tolerance of cancer cells and maintaining cancer stem cells [40,41]. Our new findings that RBP2 is critical in constitutive and inducible VEGF expression might suggest its clinical implication in gastric tumor angiogenesis and progression.

Surgery plays a central role in the overall management of operable gastric cancer. Histone deacetylase inhibitors, based on epigenetic development, are being used clinically [42], and histone demethylase inhibitors are being addressed in clinical trials [43]. Our study identified a novel molecular mechanism for RBP2 and provides better understanding of the molecular basis for angiogenetic signaling pathways, which might aid in the design of effective therapeutic modalities to control gastric cancer growth and metastasis.

## Conclusions

In this study, we detected the regulation of VEGF by H3K4 demethylase RBP2 directly *in vivo and in vitro*. This regulation plays an important role in tumorgenesis and angiogenesis of human gastric cancer. RBP2 may be critical in cancer angiogenesis.

## Materials and methods

### Patients and tissue specimens

Resected pairs of human gastric cancer tissue and distal normal gastric tissue (>5 cm from the margin of the tumor) from 27 patients were harvested during surgery at Qilu Hospital of Shandong University in 2013. No patient had received adjuvant chemotherapy before surgery. The diagnosis of gastric cancer was confirmed histopathologically. The general information for patients is in Additional file 1: Table S1. The study was approved by the ethics committee of Shandong University School of Medicine.

### Immunohistochemistry

Tissues from human resected pairs and animal models were embedded with paraffin and sliced into 5-μm pieces, which were deparaffinized and dehydrated with xylene and a graded series of alcohol. Antigen retrieval involved heat treatment performed in 0.1 M citrate buffer at pH 6.0. Then 3% H_2_O_2_ was used to block endogenous peroxidase activity. The slides were further incubated with goat serum for 30 min, then with the antibodies monoclonal rabbit anti-human RBP2 (Sigma, USA); polyclonal rabbit anti-VEGF, anti-CD31, and anti-Ki67 (all Abcam, UK); and monoclonal mouse anti-CD34 (Santa Cruz Biotechnology, USA) overnight at 4°C. The results were detected with diaminobenzidine staining (Vector Laboratories, USA) under a microscope (Olympus BX60, Tokyo) and images were captured for analysis.

### Cell lines and culture

The gastric epithelial-derived cancer cell lines BGC-823 and SGC-7901 were obtained from the cell repository for Academia Sinica (Shanghai). Human gastric epithelial immortalized GES-1 cells were maintained in our laboratory. All cell lines were grown in RPMI1640 medium (Gibco, USA) supplemented with 10% fetal bovine serum (Gibco, USA). Cell lines were incubated in a humidified atmosphere containing 5% CO_2_ at 37°C without antibiotics, then cultured on 6-well plates for 18 to 24 h before experiments.

### RNA extraction and quantitative RT-PCR

The total RNA in human or animal samples and cell lines was extracted by use of Trizol (Invitrogen, Life technologies, USA). cDNA was synthesized with use of MMLV reverse transcriptase (Fermentas, Canada). The expression of genes was checked by PCR with the TaqMan gene expression assay kit (Life Technologies, USA) or SYBR Premix Ex Taq kit (Takara, Dalian) with β-actin as a control. Real-time PCR involved the ABI7500 sequence detector (Applied Biosystems, USA). The assay IDs for human resected tissues and cell lines were RBP2, Hs00188160_m1; and VEGF, Hs00900055_m1. The PCR primers for VEGF expression in mice were sense, 5'-CTGTACCTCCACCATGCCAAGT-3', and antisense, 5'-CTTCGCTGGTAGACATCCATGA-3'. RBP2 and VEGF mRNA expression was normalized to β-actin expression. Change in expression was calculated relative to the control (2^-∆∆Ct^).

### Western blot analysis and Enzyme-linked immunosorbent assay (ELISA)

Total cellular proteins were extracted with RIPA lysis buffer. The membranes were probed with antibodies against RBP2 (Abcam, UK) and VEGF followed by anti- rabbit horseradish peroxidase-conjugated immunoglobulin G and developed by the enhanced chemiluminescent method (PIERCE, Thermo Fisher Scientific, USA). β-actin was a loading control. For immunoblotting of histone H3-K4 di- and trimethylation, we isolated a total histone fraction from nuclei using dilute acid extraction Zeng J et al., [15]. Histone proteins were detected with antibodies against di- and trimethylated H3-K4 (Abcam, UK). H3 was the control. An ELISA kit (R&D Systems, USA) was used for quantification of human VEGF secretion in cell culture supernatants.

### Promoter activity assay

The promoter sequence of VEGF was obtained from 2 databases, TRED and GENECARDS. The primer sequences for VEGF promoter amplification were sense, 5'-GGTACCTGTGAGCCTGGAGAAGTAGCC-3' (KpnI), and antisense, 5'-AAGCTTACAGTGATTTGGGGAAGTAGAGC-3' (HindIII). The result was detected by sequencing. The VEGF promoter sequence was cloned into pGL3-Basic Vector, named pGL3-VEGF. The mutation of the RBP2 binding site (CCGCCC) in the VEGF promoter sequence was preceded by PCR. The primers for mutation were sense, 5'- TGGTGGATTATAGTGGAGG -3', and antisense, 5'- CCTCCACTATAATCCACCAG -3'. The sequence was cloned into the plasmid, named pGL3-VEGF-mutant. The RBP2 expression vector and RBP2 siRNA were transfected into cells with the promoter reporter plasmids for VEGF. The Renilla luciferase-containing plasmid controlled by the thymidine kinase (TK) promoter was co-transfected as a control. Luciferase activity in the cell lysates was determined by dual luciferase reporter assay (Promega, USA) at 48 h after transfection, and the target promoter-driven firefly luciferase activity was normalized to that of TK renilla.

### Chromatin immunoprecipitation (ChIP)

Control and RBP2 siRNA-treated BGC-823 cells were cross-linked by incubation in 1% formaldehyde-containing medium for 10 min at 37°C and then sonicated to make soluble chromatin with DNA fragments between 200 and 1000 bp. Antibodies against RBP2 and tri- or di- methylated H3-K4 were used to precipitate DNA fragments bound by their corresponding elements. The protein–DNA complex was collected with protein A Sepharose beads (Millipore), eluted, and reverse cross-linked. Following treatment with protease K (Sigma-Aldrich), samples were extracted with phenol-chloroform and precipitated with ethanol. The recovered DNA was resuspended in TE buffer and used for PCR amplification. The PCR primers were for the VEGF promoter, sense, 5'- GGCGGGTAGGTTTGAATC -3', and antisense, 5'- CGTATGCACTGTGGAGTC -3'; and GAPDH, sense, 5'- AAAGGGCCCTGACAACTCTT -3', and antisense, 5'- GGTGGTCCAGGGGTCTTACT -3', as a control.

### Colony-formation assay

Cells were incubated in 6-well plates for 18 to 24 h, then transfected with the corresponding vectors for 48 h or different siRNAs for 72 h. Single cells were seeded on 6-well plates (500 cells/well). After 10 to 14 days of incubation with 2% fetal bovine serum, plates were stained with Giemsa for 20 min. Colonies with more than 50 cells were counted.

### Endothelial-cell tube-formation assay

The tube-formation assay was described previously Gong W et al., [44]. Briefly, 250 μL of growth factor-reduced Matrigel (BD Biosciences, USA) was pipetted into each well of a 24-well plate and polymerized for 30 min at 37°C. Human umbilical vein endothelial cells were harvested after trypsin treatment and suspended in conditioned medium from 1 × 10^6^ BGC-823 and GES-1-RBP2 overexpression cells or 1 × 10^6^ BGC-823 and GES-1-control cells cultured for 48 h in Dulbecco modified Eagle medium containing 1% fetal bovine serum. Then 2 × 10^4^ human umbilical vein endothelial cells in 300 μL conditioned medium were added to each well and incubated at 37°C, 5% CO_2_, for 20 h. The cultures were photographed by microscopy. The degree of tube formation was assessed as the percentage of cell surface area to total surface area. Control cell cultures values were set at 100%.

### Animal models

BGC-823 cells stably expressing RBP2 shRNA to inhibit RBP2 expression and matched controls were constructed. A total of 10 BALB/c nude mice 7 weeks old (Vital River Laboratories, P.R.China) were divided into 2 groups randomly. BGC-823 cells with RBP2 shRNA or the matched control were subcutaneously injected into mice (2 × 10^5^ cells/mouse). 15 days later, tumors were harvested and tumor size measured. Tumor volume was calculated as greatest tumor diameter × (shortest tumor diameter)^2^/2. RBP2-targeted mutant mice were provided by the Jackson Laboratory [strain B6.129S6(FVB)-Kdm5a^tm1.1Kael^/J]. The genotypes of mice were determined by protocols from the Jackson Laboratory. The primers used for PCR were for the wild type, forward, 5'- GTTTGAATTTCACTCTATGCTGGG -3'; mutant forward, 5'- GGTGCTGGGAACCATACTTG -3'; and common, 5'- TCCCAGCATGGATCTTGTCC -3'. The PCR results showed different genotypes: mutant = 530 bp, heterozygote = 216 and 530 bp, and wild type = 216 bp. All experimental protocols were approved by the local Animal Care and Use Committee.

### Statistical analysis

Quantitative data are expressed as mean ± SEM. Statistical analysis involved use of SPSS 13.0 (SPSS Inc., USA) with two-tailed Student's *t* test or one-way ANOVA for more than 2 subgroups. Statistical significance was set at *P* < 0.05.

## Competing interests

The authors declare that they have no competing interests.

## Authors' contributions

JZ, LW, LL, CC and JJ designed the study; LL, LW, PS, XG, XL, MZ and YW performed the study; LL, LW, PS, XG, XL, MZ and JZ analyzed and interpreted data; JZ supervised the study; and JZ, LW, LL, CC and JJ wrote the paper. All authors read and approved the final manuscript.

## Supplementary Material

Additional file 1: Table S1 Association of clinicopathologic variables with RBP2 and VEGF expression and MVD in human gastric cancer tissues. Click here for file

Additional file 2: Figure S2 RT-PCR results of RBP2-targeted mutant mice. Click here for file

Additional file 3: Figure S3 ELISA results for VEGF concentration in the cell cultures treated with control siRNA and RBP2 siRNA. Click here for file
