# Supplementary material for: Critical role of histone demethylase RBP2 in human gastric cancer angiogenesis
Source: Mol Cancer. 2014 Apr 9;13:81. doi: 10.1186/1476-4598-13-81 (PMC4113143; doi:10.1186/1476-4598-13-81)
Supplement: Additional file 1: Table S1 — Association of clinicopathologic variables with RBP2 and VEGF expression and MVD in human gastric cancer tissues. [file 1476-4598-13-81-S1.doc]

**Additional file 1: Table S1** Association of clinicopathologic variables with RBP2 and VEGF expression and MVD in human gastric cancer tissues.

| Category | No. of  patients | RBP2 | | | VEGF | | | MVD | | |
| --- | --- | --- | --- | --- | --- | --- | --- | --- | --- | --- |
|  |  | high | low | *P* | high | low | *P* | high | low | *P* |
| Age |  |  |  | ＞0.05 |  |  | ＞0.05 |  |  | ＞0.05 |
| < 60 years | 8 | 6 | 2 |  | 6 | 2 |  | 6 | 2 |  |
| ≥60 years | 12 | 8 | 4 |  | 9 | 3 |  | 9 | 3 |  |
| Sex |  |  |  | ＞0.05 |  |  | ＞0.05 |  |  | ＞0.05 |
| Male | 12 | 9 | 3 |  | 9 | 3 |  | 9 | 3 |  |
| Female | 8 | 5 | 3 |  | 6 | 2 |  | 6 | 2 |  |
| Differentiation |  |  |  | ＞0.05 |  |  | ＞0.05 |  |  | ＞0.05 |
| Well | 7 | 5 | 2 |  | 5 | 2 |  | 5 | 2 |  |
| Poor | 13 | 9 | 4 |  | 10 | 3 |  | 10 | 3 |  |
| Tumor size |  |  |  | <0.05 |  |  | <0.05 |  |  | <0.05 |
| ≤5cm | 10 | 5 | 5 |  | 6 | 4 |  | 6 | 4 |  |
| ＞5cm | 10 | 9 | 1 |  | 9 | 1 |  | 9 | 1 |  |
