# Supplementary figures and images for: Critical role of histone demethylase RBP2 in human gastric cancer angiogenesis
Source: Mol Cancer. 2014 Apr 9;13:81. doi: 10.1186/1476-4598-13-81 (PMC4113143; doi:10.1186/1476-4598-13-81)

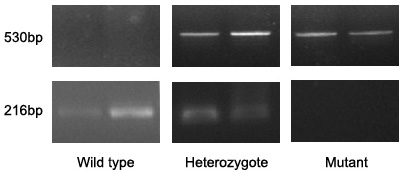

Supplement: Additional file 2: Figure S2 — RT-PCR results of RBP2-targeted mutant mice. [file 1476-4598-13-81-S2.jpeg]

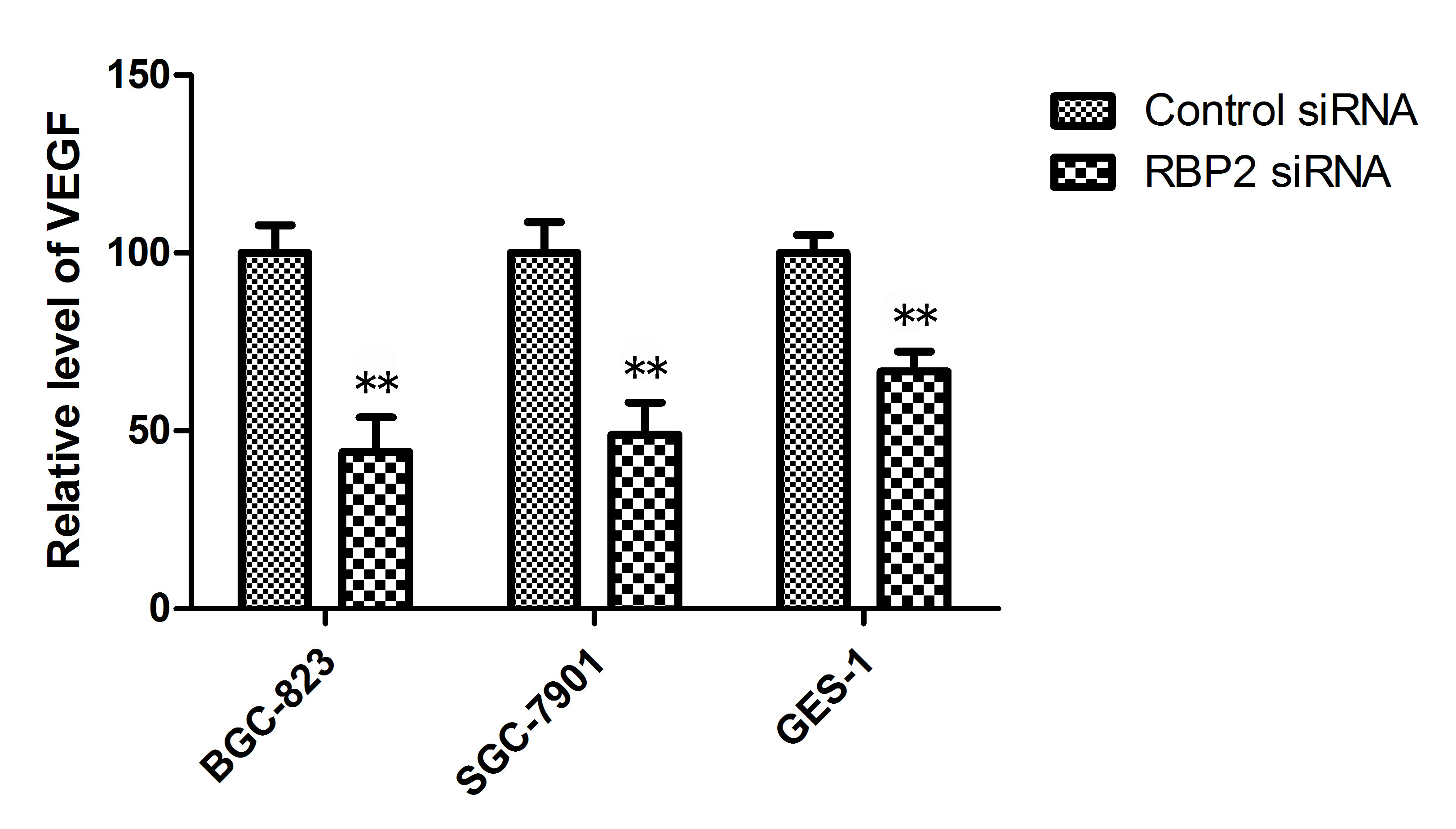

Supplement: Additional file 3: Figure S3 — ELISA results for VEGF concentration in the cell cultures treated with control siRNA and RBP2 siRNA. [file 1476-4598-13-81-S3.jpeg]
